# Commentary on: “study of the pharmacokinetic changes of tramadol in diabetic rats” is the handicapped renal pathway in DMIS contributing for the increased bioavailability of tramadol?

**DOI:** 10.1186/2008-2231-21-23

**Published:** 2013-03-15

**Authors:** Nuggehally R Srinivas

**Affiliations:** 1 Suramus Biopharm, J.P. Nagar, I Phase, Bangalore, 560078, India

## 

Dear Editor-in-Chief:

I read with interest a recently published article in your esteemed journal by Lavasani et al., entitled “Study of the pharmacokinetic changes of Tramadol in diabetic rats” [1]. Lavasani et al., have evaluated the influence of full-blown diabetes on the pharmacokinetic disposition of tramadol using a rat model of diabetes mellitus induced by streptozotocin (DMIS) [1]. Since tramadol has been independently confirmed to have glucose lowering potential in DMIS [2] and as well being investigated for its role in alleviating pain in diabetic neuropathy [3,4], this pharmacokinetic investigation in DMIS was time appropriate. The design of the study also incorporated the assessment of the role of hepatic function via a perfused liver protocol [1]. The measurements of key circulating metabolites [M1, M2 and M5] along with tramadol provided the necessary data required for the assessment of the role of diabetes on the disposition of tramadol [1].

The results of this investigation unequivocally confirmed that the exposure of tramadol increased substantially (approximately 4-fold) in diabetic rats with an identical 4-fold reduction in the oral clearance [1]. Also, the elimination rate constant for tramadol was reduced by about 1.5 fold in diabetic rats [1]. In terms of metabolites of tramadol, with the exception of M5 which showed similar exposure in diabetic vs control rats, the exposure of both M1 and M2 were about 1.42 to 1.76 fold greater in diabetic rats [1]. The perfused rat liver experiment suggested that both M1 and M5 were being formed in relatively higher amounts in diabetes rats vs control rats. Interestingly, the metabolite to parent ratio computation of diabetes rats vs control rats suggested that diabetes rats had a lower ratio in spite of higher turnover of metabolites by DMIS rat liver vs control rat liver [1]. The authors have provided interesting views to explain the observed findings of tramadol disposition in the diabetes rats that included lower volume of distribution and protein binding to account for higher tramadol exposure and possible CYP induction for the increased M1 level [1].

The intent of this note is to provide additional clues to further tease out the observed findings of tramadol disposition in diabetes rats. Although shift in volume of distribution can alter exposure, perhaps, the decreased total body clearance is the key driver to explain the exposure increase for both tramadol and the metabolite M1. The disposition of tramadol is governed by efficient hepatic clearance and renal excretion of both the parent and the major oxidative metabolites (inclusive of M1) [5]. However, in diabetes rats the renal handling system is severely handicapped [6,7] and therefore, impaired renal excretion of tramadol and M1 can lead to increased systemic exposure. It appeared that impaired renal elimination of tramadol in DMIS rat model may explain the increased bioavailability observed in diabetes rats. The impaired renal elimination would also explain the time dependent increased build-up of the tramadol exposure in diabetes rats relative to control rats (Figure one; Lavasani et al.). However, unlike the parent, the effect of renal impairment on M1 appeared to have been more pronounced post 1 h after dosing (Figure one; Lavasani et al.) because there is a lag time for the hepatic formation of the metabolite. Nevertheless, renal impairment appeared to be an important contributor for the increased exposure of M1 in diabetes rats.

In conclusion, the interesting work of Lavasani et al., has thrown some new light on the importance of well-planned experiments to tease out the variables that account for pharmacokinetic disposition of tramadol in diabetes rats [1]. The suggested work of intravenous dosing of tramadol in diabetes rats vs control rats will help in further elucidating the proposed mechanisms to explain the altered pharmacokinetic disposition of tramadol in diabetes rats [1]. However, it is strongly recommended that urinary excretion rate and renal clearance is also measured in this planned work to unequivocally establish the relationship of renal impairment on the disposition of tramadol and its metabolites in diabetes rats. Since tramadol is also excreted unchanged in humans along with the active M1 metabolite [8,9], the planned work is relevant to get dosing guidance of tramadol in patients.
